# Salvage Cryoablation for Recurrent Prostate Cancer Following Radiation—A Comprehensive Review

**DOI:** 10.3390/cancers16152717

**Published:** 2024-07-31

**Authors:** Harry Lee, Sameer Thakker, Kevin Pineault, James Wysock, Wei Phin Tan

**Affiliations:** Department of Urology, NYU Langone Health, New York, NY 10016, USA

**Keywords:** prostate cancer, focal therapy, cryoablation, cryotherapy, partial-gland ablation, radiation, prostate cancer recurrence

## Abstract

**Simple Summary:**

Apart from skin cancer, prostate cancer is the most prevalent cancer in men. The treatment options typically entail active surveillance, surgery, radiation, or a combination of the above. The treatment options for recurrent disease also include surgery, radiation, and focal therapy. In this review, we look at the use of cryoablation for recurrent prostate cancer following radiation treatment.

**Abstract:**

The treatment options for prostate cancer typically entail active surveillance, surgery, radiation, or a combination of the above. Disease recurrence remains a concern, with a wide range of recurrence rates having been reported in the literature. In the setting of recurrence, the salvage treatment options include salvage prostatectomy, salvage high-intensity focused ultrasound (HIFU), stereotactic body radiotherapy (SBRT), salvage brachytherapy, and salvage cryoablation. In this review, we analyze the currently available data related to salvage cryoablation for recurrent prostate cancer following radiation.

## 1. Introduction

Prostate cancer (PCa) is the most common non-skin cancer among men in the United States, with an estimated 191,930 new cases and 33,330 deaths in 2020 [1]. Radiation therapy is an effective treatment option for PCa and is widely used to treat PCa. The Phoenix criteria are the most accepted definition for radiation failure, and they utilize a prostate-specific antigen (PSA) rise of ≥2 ng/mL from the nadir [2]. Prior to the Phoenix criteria, the ASTRO criteria were used to determine radiation failure (three increases of PSA above the PSA nadir) [2]. The treatment options for recurrent prostate cancer following radiation include salvage cryotherapy (SCT), salvage radical prostatectomy with lymphadenectomy, salvage high-intensity focused ultrasound (HIFU), salvage brachytherapy, and salvage radiation [3]. The probability of biochemical recurrence following radiation therapy varies, with some literature citing that roughly 30–50% of patients experience biochemical recurrence within 10 years [4]. The risk of recurrence is contingent upon the initial risk group, with studies indicating that patients characterized by a higher Gleason score, elevated PSA levels, and an advanced clinical stage face an increased risk of biochemical relapse.

Cryoablation involves the use of extreme cold to destroy cancer cells [5,6,7]. Originally explored as a treatment option for primary prostate cancer, cryoablation has since shown promising results in the treatment of local recurrence after radiation therapy [8,9,10]. However, there remains a degree of uncertainty pertaining to the consensus on the use of salvage cryoablation. This review will focus on the available literature pertaining to the use of salvage cryoablation in prostate cancer recurrence.

## 2. Materials and Methods

### 2.1. Search Strategy

A literature review was conducted to assess the available literature on the effectiveness of salvage cryoablation therapy for local recurrence of prostate cancer after radiation treatment. A search of the PubMed database was conducted, using a combination of the following search terms: “salvage”, “cryoablation”, “cryosurgery”, “recurrence”, “prostate cancer”, and “radiation therapy”. The search was limited to studies with results published in English and included studies from January 1995 to December 2023. Complete search terms are listed in Supplementary Material S1.

Inclusion criteria for this review included studies that evaluated the effectiveness of salvage cryoablation therapy for local recurrence of prostate cancer after radiation treatment. Exclusion criteria included studies that did not specifically evaluate salvage cryoablation therapy, studies that did not focus on local recurrence of prostate cancer after radiation treatment, and other literature reviews.

### 2.2. Eligibility Criteria and Patients

**Figure 1:**
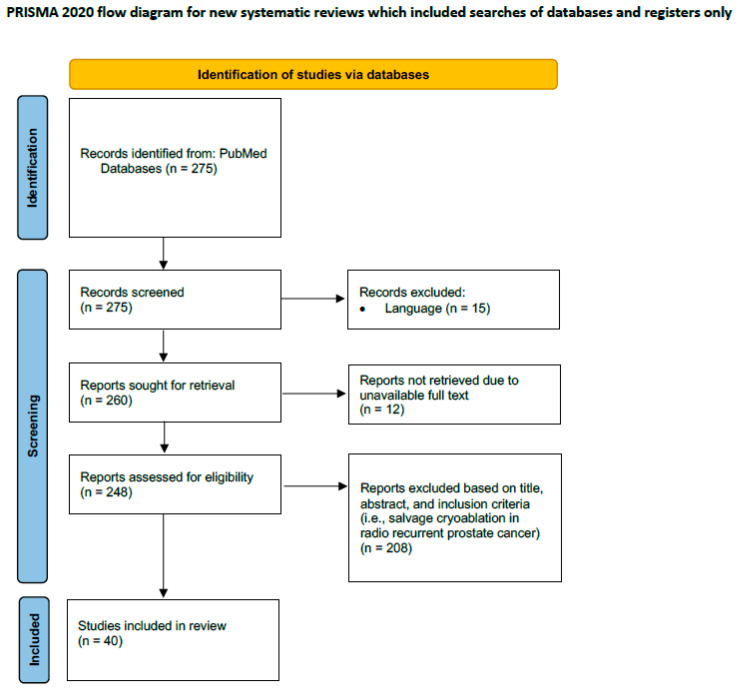
PRISMA flow diagram for study selection process.

Only articles in the English language pertaining to clinical trials involving human subjects who underwent salvage cryoablation of the prostate were included in this review. Conference abstracts, editorials, letters, case reports/case series, and review articles were excluded, although their reference lists were verified for original data (Figure 1) [11].

### 2.3. Statistical Analysis

Descriptive statistics using median and interquartile range were used to summarize demographic and baseline data of eligible patients. Sample size of individual studies and demographic values were calculated based on percentages and summed to obtain the values used for this cohort.

Data were analyzed using a narrative synthesis approach, with a focus on the overall effectiveness of salvage cryoablation therapy in the treatment of local recurrence of prostate cancer after radiation treatment. To compare survival outcomes across studies, published Kaplan–Meier plots from each trial were digitized using WebPlotDigitizer and survival probabilities and follow-up times extracted [Rohatgi A. WebPlotDigitizer Version 4.1].

## 3. Results and Discussion

### 3.1. Salvage Cryoablation Outcomes

While no randomized clinical trials exist for salvage cryoablation after radiotherapy, several studies have reported both retrospective and prospective oncologic outcomes (Table 1). The largest studies were multicenter and retrospective, with varying survival metrics. Most of the studies (58.5%) used biochemical recurrence-free survival (BRFS) or progression-free survival (PFS) as the primary outcomes, defining the treatment failure using the Phoenix criteria (PSA rise of ≥2 ng/mL from the nadir).

Some studies also reported cancer-specific survival (CSS), metastasis-free survival (MFS), and overall survival (OS). The follow-up times varied significantly, with only 21.57% (*n* = 13) of the studies reporting survival statistics for at least 5 years after cryoablation and 9.8% (*n* = 4) reporting 10-year statistics. Overall, the 5-year BRFS/PFS ranged from 43.5% to 86%, and the 10-year BRFS/PFS was 35%. The MFS at 5 years ranged from 69.4% to 100% and 79% to 86% at 10 years. The CSS ranged from 79% to 100% at 5 years and 79% to 92.5% at 10 years. The OS ranged from 73% to 100% at 5 years and 45% to 76% at 10 years.

**Figure 2:**
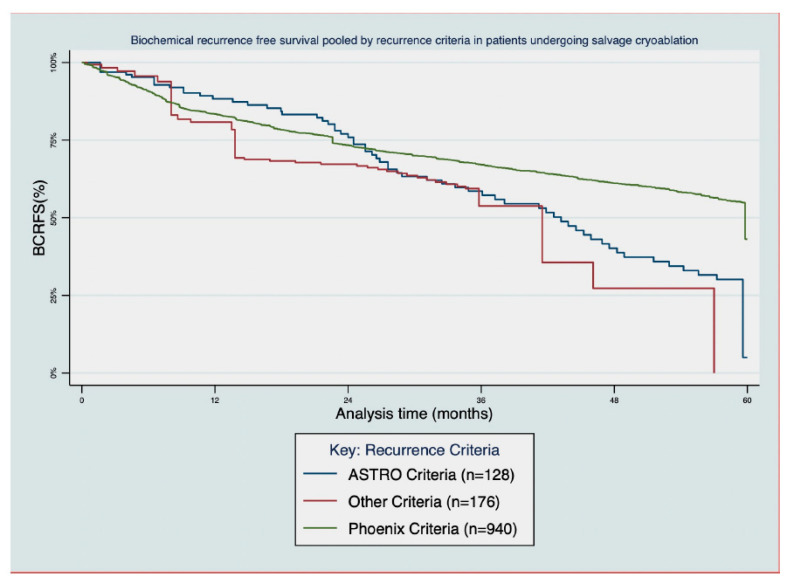
Aggregate Kaplan-Meier curve for biochemical recurrence free survival pooled by recurrence criteria.

The BRFS Kaplan–Meier curves were available in 18 studies, showing significant variability in efficacy. A notable portion of the studies showed BRFS rates of around 50% or less at 30 months, even when pooled by different recurrence criteria (Figure 2).

The morbidity data included incontinence, stricture, erectile dysfunction, rectal or fistula injury, and infection (Table 2). The incontinence rates ranged from 2.1% to 95.5%, and the rectal or fistula injury rates ranged from 0% to 9.1%.

Most of the reviewed studies had shared limitations, particularly in the imaging modalities and patient selection. Many of the studies did not incorporate CT and/or MRI or routinely utilize bone scans. The data predated the PSMA PET era, suggesting that the patient selection and outcomes could improve with regular imaging incorporation.

Overall, salvage cryoablation appears to be an effective treatment for the local recurrence of prostate cancer post-radiation therapy in carefully selected patients. Recent studies show promising cancer control and lower complication rates than earlier studies. However, imaging and patient selection limitations remain, highlighting the potential for improved outcomes with enhanced patient selection.

### 3.2. Whole-Gland Oncologic Outcomes

A total of 23 studies on salvage whole-gland cryoablation for radiation-resistant prostate cancer recurrence were published. The 5-year BRFS ranged from 45% to 86%, and the 5-year OS ranged from 74% to 100%.

All the patients had local and biopsy-proven recurrence after primary radiotherapy. The largest prospective series by Siddiqui et al. examined 157 patients (mean age 69.4; mean pre-salvage PSA 6.6 ng/mL) with a median follow-up of 117 months. They reported 10-year overall, biochemical disease-free, and metastasis-free survival rates of 76%, 35%, and 86%, respectively [36]. The second-largest cohort from MD Anderson Cancer Center evaluated 150 patients, finding a 5-year disease-free survival rate of 26% for those with prostate cancer following cryotherapy and 52% for those without. However, many patients likely had metastatic disease and did not undergo adequate imaging [22].

Smaller cohort studies with shorter follow-ups showed comparable oncologic benefits of salvage cryoablation to salvage prostatectomy after primary radiotherapy. Donnelly et al. found 51% and 44% biochemical recurrence-free rates at 1 and 2 years, respectively, using a PSA definition for a biochemical failure of PSA ≥ 0.3 ng/mL [23]. Robinson et al. found 64.1% and 51.6% recurrence-free rates at 1 and 2 years, respectively, with a similar PSA cutoff [24].

In Siddiqui et al.’s large prospective series, the pre-cryoablation and nadir PSA values were significant predictors of metastasis-free and biochemical-free survival, while the age at salvage cryoablation and nadir PSA predicted the overall survival [36]. The MD Anderson cohort found that fewer cryoprobes and freeze–thaw cycles indicated inadequate therapy [22].

The retrospective studies also showed similar oncologic benefits. A large retrospective series of 187 men reported 10-year cumulative incidences of biochemical recurrence, prostate cancer-specific mortality, metastasis, and ADT initiation of 55.8%, 21.2%, 51%, and 16.5%, respectively [47]. Smaller retrospective studies showed varying BRFS rates, with the follow-up times ranging from 22 to 39 months [18,28,31,43,48].

### 3.3. Whole-Gland Salvage Cryoablation Morbidity

Salvage treatments, while lifesaving, are associated with significant complications and morbidity. The Mayo Clinic and MSKCC/Baylor Medical Center reported high complication rates for salvage prostatectomy, including rectal injury (5%), urinary extravasation (15%), and bladder neck contracture (22%) [49,50].

The early cryoablation studies also reported high complication rates, including impotence, incontinence, and fistulas. However, the recent studies show decreased complication rates due to technological advancements, such as urethral warmers, live ultrasound, and double freeze cycles. The mild to moderate incontinence rates ranged from 9% to 95.5%, and the severe incontinence rates ranged from 3% to 5%. The LUTS varied from 15.6% to 67%, hematuria from 5% to 7.9%, and erectile dysfunction from 56% to 90% [6,10,12,13,14,16,18,31,35,45,46,51,52].

### 3.4. Focal Cryoablation

Since the establishment of the feasibility and comparable outcomes of whole-gland salvage cryoablation, there has been an increased utilization of focal gland salvage cryoablation, the hope being a further reduction in the morbidity associated with whole-gland cryoablation while maintaining similar or equivalent oncologic outcomes. Multiple studies have shown promising results, particularly in the short- to mid-range outcomes. One study looking at 385 men found no statistically significant difference in the progression-free survival rate at 2 years between whole-gland versus focal salvage cryoablation (79.8% vs. 76.98%; *p* = 0.11) [42]. A second study assessed the efficacy of salvage cryoablation in 898 patients utilizing ADT-free survival as a surrogate. The study found no statistically significant difference in the post-operative use of ADT between whole versus focal salvage cryoablation patients. The 5-year ADT-free survival of whole-gland salvage cryoablation was 71.3% and 73.1% for partial-gland cryoablation (*p* = 0.908) [38].

Additional studies have highlighted encouraging results. In a smaller study conducted on 19 patients undergoing partial-gland salvage cryoablation by Eisenberg et al., they found the biochemical recurrence-free rates to be 89%, 67%, and 50% at 1, 2, and 3 years, respectively, when using the ASTRO criteria of three consecutive PSA rises after the nadir [27]. The rates were even more promising when utilizing the Phoenix criteria, with the 1-, 2-, and 3-year biochemical recurrence rates at 89%, 79%, and 79%, respectively. These differences within the context of the different criteria used were reflected in other studies. For instance, a study looking at 100 patients who underwent focal salvage cryoablation and utilized the ASTRO criteria found the biochemical recurrence-free rates to be 83% at 12 months, 72% at 24 months, and 59% at 36 months [26], whereas another study with a sample size of 91 that utilized the Phoenix criteria found biochemical disease-free survival rates of 95.3% and 72.4% at 1 and 3 years [34].

With that said, focal gland therapy was not without its own deficits. Differences between the focal and whole-gland treatments were recognized, particularly regarding longer-term outcomes. A different study that utilized the Phoenix criteria for biochemical failure found the 5-year biochemical failure-free survival rates for focal cryoablation and total cryoablation to be 54% and 86%, respectively [3]. This pattern of a noticeably lower 5-year biochemical recurrence-free rate was also reflected in other studies, such as the one conducted by Li et al., which found a rate of 46.5% at 5 years [34].

In the setting of oncologic outcomes, the importance of careful patient selection when it comes to focal salvage cryoablation has also been underlined. For instance, a 2003 study looking at 7-year biochemical disease-free survival rates found improved rates in patients with lower pre-op PSAs. When using a PSA cutoff of 0.5, patient groups with pre-op PSAs of <4, 4–10, and >10 were found to have rates of 60.8%, 62%, and 50%. When the PSA cutoff was increased to 1, the same groups based on the pre-op PSA had rates of 78.4%, 74.3%, and 45.7% [21]. In a different study looking at 118 patients, Chin et al. found a pre-salvage cryoablation PSA > 10, a Gleason score of 8 or greater before radiation, and stage T3/4 disease to predict unfavorable biochemical outcomes in patients who underwent salvage cryoablation [17].

### 3.5. Focal Gland Salvage Cryoablation Morbidity

As previously discussed, salvage cryoablation, although less morbid than salvage prostatectomy, is still not without its own set of potential complications. Partial-gland cryoablation was subsequently presented to further decrease the associated morbidity while sustaining comparable oncologic outcomes. Various studies have since been published that have found focal cryoablation to be less morbid than whole-gland cryoablation.

Li et al. found focal salvage cryoablation with improved potency preservation compared to whole-gland salvage [34]. In another study looking at six patients who underwent whole-gland treatment and fifteen patients that underwent focal treatment, five patients had major side effects. Four of the five patients had undergone whole-gland treatment [39]. The complications in the whole-gland treatment cohort included persistent incontinence requiring an artificial urinary sphincter and chronic pelvic pain requiring a multimodal pain regimen. The complications in the focal cohort entailed urethral stenosis requiring self-catheterization.

When it comes to one of the most devastating morbidities associated with salvage cryoablation—fistulas—it can be postulated that focal ablation has a potentially lower associated risk. The overall low incidence of fistulas following cryoablation means that all the reviewed studies lack the power to show an objective benefit. However, a smaller area undergoing cryoablation suggests overall less damage to the prostatic blood supply, and thus less necrosis and risk of fistula.

With that said, some studies also failed to show any major significant differences regarding potential side effects. A study by Tan et al. found focal salvage cryoablation to only be associated with a lower probability of post-procedural urinary retention compared to whole-gland cryoablation (5.6% vs. 22.4%; *p* < 0.001) [42]. The study found no significant differences in the rates of rectal fistula, urinary incontinence, or erectile dysfunction [42]. Abreu et al. also noted that, although the patients that underwent focal cryoablation in their study had a lower number of individuals who developed treatment-related morbidity (e.g., incontinence, erectile dysfunction, or rectourethral fistula) relative to whole-gland cryoablation, the differences were not statistically significant [3].

## 4. Conclusions

Salvage cryoablation for locally recurrent prostate cancer following radiation is a viable option in carefully selected patient populations. The rates of cancer control remain promising, and the complication rates have only continued to improve with the introduction of better technology and modifications in technique.

## Figures and Tables

**Table 1:** Survival rates associated with salvage cryoablation for recurrent prostate cancer.

Authors	Year	Study Type	Focal vs Whole Gland	Follow Up Period	N	BFS	MFS	CSS	OS
Bales et al. [12]	1995	Prospective, phase II trial	Whole	12–23 months	23	12 months: 18%; 17 months: 11%	64%	100%	95.70%
Miller et al. [13]	1996	Retrospective review	Not specified	16.8 months	33	35 months (PSA < 0.4): 19%	90.90%	-	-
Pisters et al. [14]	1997	Phase I/II trial	Not specified	13.5 months	150	Average 13.5 months: 42%	-	-	-
Benoit et al. [15]	2000	Retrospective review	Whole	5 years	87	5 years: 69.4%	69.40%	-	-
De La Taille et al. [16]	2000	Retrospective review	Whole	21.9 month	43	6 months: 79%; 12 months: 66%	100%	-	-
Chin et al. [17]	2001	Retrospective review	Focal	18.6 months	125	30 months: PSA >4–68%, >2–55%, >0.5–34%	91.50%	-	-
Ghafar et al. [18]	2001	Retrospective review	Whole	20.7 months	38	12 months: 86%, 24 months: 74%	-	-	-
Zisman et al. [19]	2001	Retrospective review	Whole	-	92	-	-	-	-
Izawa et al. [20]	2002	Prospective study	Not specified	4.8 years	131	5 years: 40%	-	5 years: 79%	5 years: 73%
Bahn et al. [21]	2003	Retrospective review	Focal	82.3 months	59	7 years: using 0.5 PSA cutoff/pre-op PSA <4–60.8%, 4—10–62%, >10–50%; Using 1.0 PSA cutoff / pre-op PSA < 4–78.4%, 4—10–74.3%, >10–45.7%	100%	100%	100%
Izawa et al. [22]	2003	Prospective study	Not specified	>6 months	150	5 years for patients wth PCa on follow up biopsy: 26%; 5 years for patients without PCa on follow up biopsy: 52%	-	-	-
Donnelly et al. [23]	2005	Prospective, phase II trial	Whole	20 months	46	1 year: 51%; 2 year: 44%	94%	100%	100%
Robinson et al. [24]	2006	Prospective, phase II trial	Not specified	2 years	46	12 months: 64%, 24 months: 52%	-	97.80%	93.50%
Gowardhan et al. [25]	2007	Prospectively collected data, retrospective review	Whole	36 months	42	1 year: 61%	-	-	-
Ismail et al. [26]	2007	Prospective case series	Not specified	33.5 months	100	12 months: 83%, 24 months: 72%, 36 months: 59%	-	-	-
Eisenberg et al. [27]	2008	Retrospective review	Focal	18 months	19	ASTRO criteria—1 year: 89%, 2 years: 67%, 3 years: 50%; Phoenix criteria—1 year: 89%, 2 years: 79%, 3 years: 79%	43%	-	-
Pisters et al. [28]	2008	Retrospective review	Not specified	21.6 months	279	ASTRO criteria—5 years: 58.9%; Phoenix criteria—5 years 54.5%	-	-	-
Cheetham et al. [29]	2010	Retrospective review	Whole	10.1 years	76	-	86.80%	10 year: 87%	10 years: 56.6%
Spiess et al. [30]	2010	Retrospective review	Not specified	3.4 years	450	Median 3.4 years: 34%	-	-	-
Abreu et al. [3]	2013	Prospectively collected data, retrospective review	Both	53 months	50	5 years: focal—54%, whole gland—86%	98%	100%	100%
Peters et al. [31]	2013	Retrospective review	Not specified	14 months	54	14 months: 39%	-	100%	91%
Spiess et al. [32]	2013	Retrospective review	Not specified	3.8 years	156	1 year: 89%, 2 years: 73.7%, 3 years: 66.7%	-	-	-
Wenske et al. [33]	2013	Retrospective review	Both	47.8 months	328	5 years: 63%, 10 years: 35%	5 years: 89%, 10 years: 79%	5 years 91%, 10 years 79%	5 years 74%, 10 year 45%
Li et al. [34]	2015	Retrospective review	Focal	15 months	91	1 year: 95.3%, 3 uears: 72.4%, 5 years: 46.5%	-	-	-
Lian et al. [35]	2016	Retrospective review	Not specified	63 months	32	5 years: 43.5%	100%	5 years: 100%	5 years: 92.3%
Siddiqui et al. [36]	2016	Prospective study	Whole	117 months	187	5 years: 45%, 10 years: 35%, 15 years: 22.6%	10 years: 86%, 15 years: 71%	92.50%	5 years: 93%, 10 years: 76%
Overduin et al. [37]	2017	Retrospective review	Focal	24 months	47	-	79%	-	-
Ginsburg et al. [38]	2017	Retrospective review	Both	19 months	898	Median time 13.4 months: 23.7%	-	-	-
Barat et al. [39]	2019	Retrospective review	Both	20 months	28	2 years: 65.5%	92.90%	-	92.90%
Bomers et al. [40]	2019	Retrospective review	Focal	>12 months	62	6 months: 83%; 12 months: 63%	90.30%	-	98.40%
Safavy et al. [41]	2019	Retrospective review	Both	3.9 years	75	3.9 years: 50.7%	-	-	-
Tan et al. [42]	2019	Retrospective review	Both	24.4 months	385	Median 24.4 months: 78.3%	-	-	-
Bain et al. [43]	2020	Retrospective review	Whole	56.1 months	37	2 years: 71%	82%	-	-
Bauman et al. [44]	2020	Retrospective, propensity matched analysis	Whole	18 years	169	-	-	83.80%	12.33 years
Nair et al. [28]	2020	Retrospective review	Whole	25.1 years	186	-	-	75.50%	18.4%, 11.8 years
Tan et al. [5]	2021	Retrospective review	Focal	12 months	11	12 months: 100%, 24 months: 80%, 36 months: 40%	12 months: 100%, 24 months: 75%, 36 months: 50%	-	-
Campbell et al. [10]	2023	Retrospective review	Both	72 months	419	2 years: 86.9%; 5 years 78.5%	-	-	-
Chin et al. [45]	2023	Prospectively collected data, retrospective review	Whole	149 months	187	12 years: 36%	12 years: 78%	12 years: 81%	12 years: 56%
Tan et al. [9]	2023	Prospectively collected data, retrospective review	Whole	71 months	110	2 years: 81%; 5 years 71%	-	-	-
Ramalingam et al. [46]	2023	Retrospective review	Both	10 months	18	10 months: 88.9%	-	-	-

**Table 2:** Reported morbidities associated with salvage cryoablation for recurrent prostate cancer.

Authors	Year	N	Incontinence, %	Stricture	Erectile Dysfunction, %	Rectal/Fistula Injury	Venous Thromboembolism	Infection	Blood Transfusion
Bales et al. [12]	1995	23	95.50%	13.60%	100%	-	-	64%%	-
Miller et al. [13]	1996	33	10.30%	5.10%	-	0%	-	15.40%	-
Pisters et al. [14]	1997	150	73%	-	72%	0.60%	-	-	0%
Benoit et al. [15]	2000	87	13.80%	6.60%	-	0%	-	0.60%	-
De La Taille et al. [16]	2000	43	9%	4.70%	-	0%	-	9%	-
Chin et al. [17]	2001	125	20.30%	1.60%	-	3.30%	-	-	-
Ghafar et al. [18]	2001	38	7.90%	-	-	0%	-	2.60%	-
Zisman et al. [19]	2001	92	3.30%	-	-	0%	-	0%	-
Izawa et al. [20]	2002	131	-	-	-	-	-	-	-
Bahn et al. [21]	2003	59	4.30%	-	-	3.40%	-	-	-
Izawa et al. [22]	2003	150	-	-	-	-	-	-	-
Donnelly et al. [23]	2005	46	6.50%	-	100%	2.10%	-	-	
Robinson et al. [24]	2006	46	-	-	-	-	-	-	-
Gowardhan et al. [25]	2007	42	-	-	100%	7.10%	-	-	-
Ismail et al. [26]	2007	100	13%	-	86%	1%	-	-	-
Eisenberg et al. [27]	2008	19	5.30%	5.30%	60%	-	-	-	-
Pisters et al. [28]	2008	279	5.80%	-	-	1.20%	-	-	-
Cheetham et al. [29]	2010	76	-	-	-	-	-	-	-
Spiess et al. [30]	2010	450	-	-	-	-	-	-	-
Abreu et al. [3]	2013	50	6%	-	81.8% of patients with erectile function prior to cryoablation	2%	-	-	-
Peters et al. [31]	2013	54	-	-	93%	7%	-	-	-
Spiess et al. [32]	2013	156	-	-	-	-	-	-	-
Wenske et al. [33]	2013	328	2.10%	4.60%	-	1.80%	-	-	-
Li et al. [34]	2015	91	5.50%	-	50%	3.30%	-	-	-
Lian et al. [35]	2016	32	12.50%	-	57.10%	-	-	3.10%	-
Siddiqui et al. [36]	2016	187	39.60%	7%	-	2.50%	-	10.20%	-
Overduin et al. [37]	2017	47	-	-	-	-	-	-	-
Ginsburg et al. [38]	2017	898	-	-	-	-	-	-	-
Barat et al. [39]	2019	28	10.70%	3.60%	-	-	-	-	-
Bomers et al. [40]	2019	62	3.20%	-	-	4.80%	-	9.70%	-
Safavy et al. [41]	2019	75	25.30%	6.70%	-	2.70%	1.30%	-	-
Tan et al. [42]	2019	385	14%	-	58.40%	-	-	-	-
Bain et al. [43]	2020	37	-	10.80%	-	-	-	2.70%	-
Bauman et al. [44]	2020	338	-	-	-	-	-	-	-
Nair et al. [28]	2020	186	-	-	-	-	-	-	-
Tan et al. [5]	2021	11	9.10%	-	-	9.10%	-	-	-
Campbell et al. [10]	2023	419	16%	-	85.90%	2.60%	-	-	-
Chin et al. [45]	2023	187	3.70%	-	-	3.70%	-	-	-
Tan et al. [9]	2023	110	9%	-	-	-	-	-	-
Ramalingam et al. [46]	2023	18	5.56%	5.56%	-	5.56%	-	-	-

## Data Availability

Not applicable.

## Supplementary Materials

The following supporting information can be downloaded at https://www.mdpi.com/article/10.3390/cancers16152717/s1, Supplementary Material S1: Search criteria in PubMed.
