# Supplementary material for: Salvage Cryoablation for Recurrent Prostate Cancer Following Radiation—A Comprehensive Review
Source: Cancers (Basel). 2024 Jul 31;16(15):2717. doi: 10.3390/cancers16152717 (PMC11312114; doi:10.3390/cancers16152717)
Supplement: Supplementary file 1 [file cancers-16-02717-s001.zip › cancers-3078575-supplementary.pdf]

Supplementary Material S1

Search criteria in PubMed:

(((((((“Salvage cryoablation” “radiation therapy”) OR (“Salvage” “cryoablation” “radiation therapy”)) OR (“Salvage cryoablation” “local recurrence”)) OR (“Salvage” “cryoablation” “prostate cancer”)) OR (“Salvage” “cryoablation” “prostate” “recur-rent”)) OR (“Salvage” “cryotherapy” “prostate” “recurrent”)) OR (“Recurrent” “cryosurgical ablation” “prostate”)) OR (“Salvage” “cryosurgery” “prostate”).
